# Deductive Falsification Instead of Inductive Verification As Logical Basis for the Critical Appraisal of Randomised Controlled Trials

**DOI:** 10.7759/cureus.81397

**Published:** 2025-03-29

**Authors:** Steffen Mickenautsch, Veerasamy Yengopal

**Affiliations:** 1 Faculty of Dentistry, University of the Western Cape, Cape Town, ZAF; 2 Community Dentistry, School of Oral Health Sciences, Faculty of Health Sciences, University of the Witwatersrand, Johannesburg, ZAF

**Keywords:** bias identification, clinical trial appraisal, critical reasoning, randomised clinical trial, systematic review and meta analysis

## Abstract

Randomised controlled trials (RCTs) aim to rigorously examine the cause-and-effect relationship between disease treatment and its clinical outcome. The success of this endeavour depends on the absence of errors in the applied RCT methodology. To identify potential errors, RCTs undergo critical appraisal using trial appraisal tools. Currently, the most recommended tool for assessing the risk of systematic error (bias) in RCTs is the second version of Cochrane’s Risk of Bias tool (RoB 2; Cochrane, London). This review shows that the application of the RoB 2 tool is based on inductive verificationist reasoning, which leads to invalid circular inferences or a lack of justification for RCT result validity. More importantly, inductive verification also allows formal logical justification that RCTs with an overall ‘low risk of bias’ rating do not require critical re-appraisal in the future. This poses a practical risk, preventing the re-appraisal of high-bias risk RCTs that were erroneously rated as ‘low risk of bias’ in the past and allowing the conclusions of such RCTs to continue guiding clinical practice. In contrast, deductive falsificationist reasoning is free from these shortcomings and may provide a more robust logical basis for the critical appraisal of RCTs. Classical critique of deductive falsification is insufficient for dismissing it as a basis for RCT appraisal.

## Introduction and background

Randomised controlled trials (RCTs) aim to rigorously examine the cause-and-effect relationship between disease treatment and its clinical outcome [1]. The realisation of this objective depends on the absence of systematic and random errors in the applied RCT methodology. To identify potential errors, RCTs undergo critical appraisal, typically during the systematic reviews of clinical trials, using pre-specified appraisal criteria provided by specialised trial appraisal tools. The currently most recommended appraisal tool for assessing the risk of systematic error (bias) in RCTs is the second version of Cochrane’s Risk of Bias tool (RoB 2; Cochrane, London) [2].

Using the RoB 2 tool, RCT characteristics are appraised in five specific bias domains: bias arising from the randomisation process; bias due to deviations from intended interventions; bias due to missing outcome data; bias in measurement of the outcome, and bias in selection of the reported results. During the appraisal process, a total of 22 questions are answered with one of three response options: ‘lower’ and ‘higher risk of bias’, and ‘other’. The entire RCT is judged as of overall ‘low risk of bias’, when the ‘low risk of bias' response option is assigned to all of its five bias domains, indicating that the trial is at low risk of bias for all domains [2].

Induction is a type of reasoning that generalises from a limited number of observations and formulates universal statements as a result [3]. By judging a trial as of overall ‘low risk of bias’ using the RoB 2 tool, a positive universal statement is inductively made from the limited number of 22 question answers, concerning the limited number of five bias domains, about the nature of all possible trial characteristics. Such a universal statement is confirmed (= verified) by the ‘low risk of bias’ judgement for all of five bias domains.

By using the RoB 2 tool, the RCT is not judged to be ‘bias free’ but rather to have a ‘low risk of bias’. Hence, no definitive statement is made regarding the existence of bias. Instead, the RoB 2 tool refers to the ‘risk’ of such existence [2]. There is no consensus on the meaning of ‘risk’ [4]. However, within the context of Cochrane’s RoB 2 tool, ‘risk’ is defined by the Cochrane Handbook for Systematic Reviews of Interventions as the ‘extent’ to which bias may have affected trial results [5]. Thus, ‘risk’ is not linked to any consideration of probability or probabilistic stance traditionally associated with the term.

Deduction is a type of reasoning that infers specific conclusions from general statements, while falsification is the negation of those statements [3]. In the context of trial appraisal, deductive falsificationist reasoning does not confirm the validity of RCT results or the extent of bias affecting them as ‘low’. Instead, it only identifies instances where results are invalid or biased. No positive universal statement about trial validity is generated from the appraisal process based on limited observations, and no assumptions are made concerning trial characteristics that are outside any applied appraisal criteria. Instead, the universal statement that RCT results are valid (or that the extent of bias affecting trial results is ‘low’) is provisionally accepted as conjecture or hypothesis. The subsequently applied appraisal process tests this hypothesis.

Depending on the appraisal outcome, the universal statement as a hypothesis is either falsified or corroborated. Hypothesis falsification indicates that RCT results are invalid (or that the extent of bias affecting trial results is ‘high’). Corroboration does not confirm the hypothesis or provide the basis for its acceptance. It only indicates that the trial has complied so far with all appraisal criteria, without giving any assurance that trial validity may not be falsified during any further appraisal according to other criteria or test methods. In that way, deductive falsification encourages the re-appraisal of RCTs that were corroborated in the past.

The objectives of this review are to demonstrate that RCT appraisal using the RoB 2 tool follows inductive verificationist reasoning, to highlight the associated logical problems and practical dangers, and to argue that deductive falsification offers a superior alternative for critical RCT appraisal.

## Review

The RoB 2 relies on a limited number of question answers (22) across a limited number of bias domains (5) to make a judgement about the nature of all possible trial characteristics (N_TC_). The total number of possible trial characteristics (N_TC_) can reasonably be assumed to be finite but, for practical purposes, unknowable. N_TC_ represents the sum of the number of characteristics reported in the appraised RCT (N_A_); the number of all characteristics reported in the RCT that were outside the scope of the five bias domains and thus were not appraised by the RoB 2 tool (N_B_) and the number of all trial characteristics generated during trial conduct but not included in the appraised RCT report (N_C_).

Any judgement based on RoB 2 appraisal results combines empirical observations (N_A_) with the assumption that all other trial characteristics (N_B_ and N_C_) also meet the criteria of ‘low risk of bias’. However, this assumption is based on limited empirical observations and the further assumption that these observations are representative of all, leading to an infinite regress, as well as an invalid inference, a phenomenon known as ‘naïve-inductivism’. Further details about the nature of such infinite regress and naïve inductivism in general have been described elsewhere [3,6]. The inference is invalid because the ‘low risk of bias’ results from the appraised bias domains cannot empirically support the assumption that non-appraised trial characteristics (N_B_ and N_C_) have a low extent of bias. Study results support this. In a simulation study, 45 trials were generated and randomly assigned to zero to five errors out of a total of 65 error domains that may affect clinical evidence and which, so far, were identified by the Bias Collaboration [7]. The trials were then appraised for errors with a simulated appraisal tool consisting of five pre-specified error domains. From the appraisal results, the negative likelihood ratio (-LR) with 95% confidence interval (CI) was computed. The -LR was 0.84 (95% CI: 0.80 - 0.88), suggesting that error-free evidence is only 1.2 times more likely to be rated as ‘low bias risk’/‘high-quality’ than evidence containing some form of error [8].

The naïve-inductive generalisation of an overall ‘low risk of bias’ judgement from a limited number of observations may be only one possible interpretation of RoB 2 trial appraisal results for an RCT. Alternatively, the overall judgement could be viewed as a summary report of the observations made during trial appraisal, referring solely to the five appraised RoB 2 bias domains without making any generalisation beyond. However, such a radically empirical interpretation represents a strict positivist stance [6] and provides no judgement about the overall (internal) validity of the reported RCT results. From this perspective, no knowledge can be gained about the extent to which systematic error (bias) may have affected the actual treatment effect estimate, that is, whether the risk is low or high.

From the demonstrated problems related to naïve-inductivism and strict positivism, it can be concluded that an overall ‘low risk of bias’ rating of an RCT by use of Cochrane’s RoB 2 tool provides no logical justification for any certainty that the reported RCT results are valid and thus fit for clinical guidance. However, it can be argued that absolute certainty is unattainable in real-world situations, and a pragmatic approach would be to prefer results from ‘low risk of bias’ rated RCTs over those from ‘higher risk of bias’ rated RCTs. While this approach is rational and correct, it poses a practical risk: future critical re-appraisal of ‘low risk of bias’ rated RCTs would lack logical justification. Such lack of justification can be inferred as follows: Let ‘A’ be the universal statement that an RCT is overall of ‘low risk of bias’ and ‘B’ the statement of any future single ‘low risk of bias’ re-appraisal result. By affirmation of the antecedent ‘A’, according to the Modus ponens rule of propositional logic (((A -> B). A) -> B) [9], it can correctly be argued that if ‘A’ is the case then ‘B’ is the case (A -> B). Because ‘A’ is always confirmed (i.e., it has been verified by the RoB 2 tool), then any ‘->B’ is necessarily always confirmed, too. Hence, (at least as long as the RoB 2 is not modified or updated) any future re-appraisal of the RCT lacks logical justification.

Such a logical stance may even be empirically supported. For example, trial authors often conduct statistical significance testing of baseline variables in RCTs, in order to verify the effectiveness of the randomisation process and therefore the trial’s low risk of selection bias. A negative result of such a test confirms the overall RoB 2-based judgement of the trial and therefore affirms that any re-appraisal of bias risk in RCTs beyond the application of the RoB 2 tool was unnecessary.

However, it has been shown that statistical significance testing of baseline variables in RCTs can lead to misleading conclusions, due to the test’s high rate of false negative results [10]. When 1,070 RCTs were re-examined for selection bias using the more accurate ‘simulated comparator trial’ (SCT) adjusted version of the I^2^ test [11] instead, the results revealed a 6% higher likelihood for high selection bias risk in all RCTs that were previously rated as of ‘low bias risk’ with the RoB 2 tool than RCTs with high-risk rating (Negative likelihood ratio 1.06; 95% Confidence interval: 0.98 - 1.15) [12]. Based on the lack of logical justifiability for re-appraising RCTs with low-bias risk rating, the re-appraisal with the more accurate (SCT adjusted) I^2^ - test would have been omitted, and the actual high-bias risk RCTs not identified.

The non-suitability of RCT results with high bias risk for clinical guidance underscores the practical danger that a lack of logical justification for critically re-appraising any ‘low risk of bias’ rated RCT in the future by use of new, more accurate tests concerning current bias domains or appraisal in any additional domains poses.

Because the RoB 2 tool relies on inductive verificationist reasoning, it leads to invalid circular inferences (naïve-inductivism) or alternatively fails to justify trial result validity (strict positivism). Moreover, this type of reasoning provides a logical basis for concluding that overall ‘low risk of bias’ rated RCTs do not require future re-appraisal. This carries the practical danger that results from actual high-bias risk RCTs that were erroneously rated in the past as ‘low risk of bias’, will continue to guide clinical practice.

In contrast, trial appraisal based on deductive falsification relies on the asymmetry between verification and falsification. The asymmetry lies in the fact that universal statements, such as the overall validity of reported trial results, cannot be verified from single observations, regardless how many appraisal criteria a trial has complied with, but can at any time be contradicted by one single observation (falsified) that shows a systematic error in any one bias domain [12]. This asymmetry is logically justified by the denial of the consequent ‘B’, according to the Modus tollens rule of propositional logic [9]. Specifically, let ‘A’ be the universal statement ‘the entire trial is at low risk of bias’ and ‘B’ be the statement ‘any future single re-appraisal result indicates low risk of bias’. The hypothesis to be tested will be: if ‘A’ is the case, then ‘B’ is the case (A -> B). If during trial re-appraisal a ‘high risk of bias’ in one single trial characteristic (-B) is established, then ‘A’ is falsified (-A) and the entire trial regarded as being of ‘high risk of bias’ (((A -> B). -B) -> -A).

In practice, falsification during trial appraisal has been implemented, for example, by using tools like the Composite Quality Score (CQS-2B). This appraisal tool assesses trials based on four specific criteria. Trials are rated per criterion and assigned a 1-score if in compliance with the criterion, signifying corroboration, and a 0-score if non-compliant, signifying falsification for that criterion. All single scores are multiplied to an overall score, thus leading to an overall 0-score based on one single zero-rated criterion, regardless of the number of 1-scores, thus mathematically translating the asymmetry between verification and falsification into practice [13].

Deductive falsification avoids circular inference by refraining from non-empirical assumptions. Instead, it logically deduces the falsity of universal statements from the truth of singular statements, providing a clear interpretation of the actual (non-)validity of RCT results. This approach sidesteps the pitfalls of naive inductivism, and strict positivism associated with inductive verificationist reasoning in trial appraisal. By withholding overall ‘low risk of bias' verification judgements for corroborated trials and removing logical barriers, deductive falsificationist reasoning encourages future re-appraisal (and potential falsification) of previously corroborated RCTs. The distinct characteristics of deductive falsificationist and inductive verificationist reasoning are summarised in Table 1.

**Table 1:** Differences between types of reasoning

Characteristic of reasoning		Type of reasoning	
		Inductive verification	Deductive falsification
Basis	Empirical observations (appraisal results per bias/error domain)	Limited number of verifying observations	One single falsifying observation
	Non-empirical assumptions included	Yes	No
Logical rule		None	Modus tollens
Outcome (concerning overall trial validity)		Universal statement	Singular conclusion
Problems related to reasoning characteristics		Risk of naïve inductivism or	None
		Risk of strict positivism	
		Logical justification (Modus ponens) against the re-appraisal of previously verified trials possible	
Encourages trials to be re-appraised in future		No	Yes

Notwithstanding, deductive falsificationist reasoning has faced criticism on several levels [14], some of which seem relevant for its application in clinical trial appraisal. Specifically, because corroboration does not assure that trial validity will not be falsified in future appraisal, it has been argued that this approach undermines confidence in the effectiveness of any clinical treatment [15]. Furthermore, from a logical perspective [16], it has been argued that corroborated RCTs cannot be justified as preferable to falsified ones. Regarding the first critique, it's essential to consider that, contrary to common belief, no clinical evidence can provide absolute confidence in treatment effectiveness. Instead, evidence can only justify confidence in the non-validity of reported trial results. In response to the second critique, while neither falsified nor corroborated trials offer absolute certainty, it remains rational to prefer the corroborated trial evidence. This is because it has so far best survived critical appraisal and thus appears to be the better source for information than the falsified trial evidence [14]. From this perspective, it may seem that no actual difference exists between corroboration and verification of trial validity. However, verification still differs from corroboration in that it enables the application of the Modus ponens rule, which provides coherent logical justification for not re-appraising any previously verified trials.

Unlike trial verification, trial falsification during the appraisal process provides a basis for confidence in the non-validity of reported trial results, due to the falsification/verification asymmetry [12]. From an epistemological perspective, it has been argued that this confidence requires the acceptance of the applied trial falsification method (e.g., deductive falsification-based methods like CQS-2B [13]) as being infallible, either on naive or dogmatic conventional grounds. Moreover, past falsification implies that future falsification attempts will also be successful [17-19]. However, this is not the case. Every falsification method is fallible and may generate false positive results either due to errors made during the falsification procedure (measurement errors) or due to errors related to the procedure or test itself (systematic errors). This means within the context of trial appraisal that a previously falsified trial may, by repetition of the falsification method or by use of another method in the same bias domain, prove to be corroborated. Because of the obvious fallibility of falsification methods, such methods are necessarily open to falsification, themselves [14]. However, the occurrence of measurement and/or systematic errors is not specific to falsification but also possible during verification. It is important to note that if one assumes the ideal case of complete freedom from error for both, then verification still remains affected by the problems of naïve inductivism or strict positivism, and falsification is not.

Finally, the existence of the falsification/verification asymmetry has been challenged by asserting that both have in fact logical equivalence [20]. This critique is based on the convertibility of negative universal statements (for example: ‘This trial is not free of high bias risk for all bias domains.’) into positive existential statements (e.g., ‘In this trial, there exists at least for one bias domain high risk of bias.’). It has been observed that positive existential statements cannot be falsified, as a single positive observation can verify them, regardless of the number of contradictory observations. This has been presented as evidence for the equivalence between the falsification of universal statements and the verification of existential statements [20]. Nevertheless, such assertions do not negate the falsification/verification asymmetry but only mirror it. While it is correct that the logical impossibility of falsifying an existential statement is analogous to the impossibility of verifying a universal statement, a fundamental asymmetry remains between one singular sufficient observation versus an infinite number of insufficient observations to verify/falsify an existential/universal statement, respectively [14].

## Conclusions

This review has demonstrated that Cochrane’s RoB 2 tool relies on inductive verificationist reasoning, leading to either invalid circular inferences or an inability to justify certainty in the validity of ‘low risk of bias’ RCT results. Moreover, inductive verification provides formal logical justification against re-appraisal of previously verified overall ‘low risk of bias’ rated clinical trials. In contrast, we argue that deductive falsificationist reasoning is free of such shortcomings and thus may provide a better basis for the critical appraisal of RCTs. Classical criticisms of deductive falsificationist reasoning that are relevant to trial appraisal have been shown to be insufficient for rejecting deductive falsification on logical grounds.
